# Pregnant women’s preference and factors associated with institutional delivery service utilization in Debra Markos Town, North West Ethiopia: a community based follow up study

**DOI:** 10.1186/s12884-015-0437-z

**Published:** 2015-02-05

**Authors:** Hinsermu Bayu, Mulatu Adefris, Abdella Amano, Mulunesh Abuhay

**Affiliations:** Department of Midwifery, College of Health Sciences, Mekelle University, Mekelle, Ethiopia; School of Medicine, Department of Obstetrics and Gynecology, College of Medicine and Health Sciences, University of Gondar, Gondar, Ethiopia; Department of Midwifery, College of Medicine and Health Sciences, University of Gondar, Gondar, Ethiopia

## Abstract

**Background:**

Majority of deaths from obstetric complications are preventable. But every pregnant woman face risks which may not always be detected through the risk assessment approach during antenatal care (ANC). Therefore, the presence of a skilled birth attendant in every delivery is the most critical intervention in reducing maternal mortality and morbidity. In Ethiopia the proportion of births attended by skilled personnel, is very low, even for women who have access to the services.

**Methods:**

A community-based follow up study was conducted from January 17, 2012 to July 30, 2012, among 2^nd^ and 3^rd^ trimester’s pregnant women in Debre-Markos town, east Gojam Zone, Amhara Region, North West Ethiopia. Simple random sampling technique was used to get a total sample size of 422 participants.

**Results:**

A total of 393 pregnant women were included in the study. The study revealed that 292(74.3%) of the pregnant women planned to deliver in a health institution. Of these 292 pregnant women 234 (80.14%) actually delivered in a health facility.

Age range from 15–19 year (AOR = 4.83, 95% CI = 1.562-12.641), college and above education of the pregnant women (AOR = 12.508, 95% CI = 1.082-14.557), ANC visit during the current pregnancy (AOR = 1.975, 95% CI = 1.021-3.392),perceived susceptibility and severity of pregnancy and delivery complication (AOR = 3.208, 95% CI = 1.262-8.155) and intention (preference) of pregnant women for place of delivery (AOR = 7.032, 95% CI = 3.045-10.234) are predictors of institutional delivery service utilization.

**Conclusions:**

Preference for institutional delivery is low in the study area. Sociodemographic factors, perception about delivery complication, ANC follow up and their intentions for institutional delivery are among important predictors of institutional delivery.

## Background

Maternal mortality has been reduced globally, but regional and country burden still remains very high [1]. Despite this recent progress, approximately 358,000 maternal deaths occurred worldwide in 2008. This means every day women die due to pregnancy and child birth related causes. Sub-Saharan Africa (SSA) and South Asia contribute about 87% to global maternal deaths. On the other hand, the lifetime risk of maternal death among regions shows that, women in sub-Saharan Africa face the highest lifetime risk of 1 in 31 [2]. Maternal mortality and morbidity levels in Ethiopia are among the highest in the world. Its Maternal Mortality Ratio (MMR) of 676 per 100,000 live births contributes about 50% to global burdens together with only five other countries namely: India, Nigeria, Pakistan, Afghanistan, and the Democratic Republic of Congo. Millennium Development Goal 5 (MDG 5) aims to improve maternal health by reducing MMR by at least 75% between 1990 and 2015. Although there is significant progress in all developing regions, the average annual percentage decline of Maternal Mortality Ratio (MMR) in sub-Saharan Africa, of which Ethiopia contributes considerably, is only 1.7%. This is very much lower than the Millennium Development Goal (MDG) target of 5.5% [1,2]. Even though a vast majority of deaths from obstetric complications are preventable, every pregnancy faces some risk which may not always be detected through the risk Assessment approach during ANC. So the best way to assure a safe and successful delivery outcome remains to be ensuring the presence of a skilled birth attendant in every childbirth. The presence of a skilled birth attendant during delivery is one of the indicators used in achieving MDG5. Surprisingly, in Ethiopia the proportion of births attended by skilled personnel is very low, only 10%, even in areas where women have access to the services [3]. However, much it is not known why majority of pregnant women in the study area and the country as a whole do not deliver their babies in a health institution. Some of the studies done before are cross –sectional with the recall history of as long as five year/s back. So that the identification of factors associated with institutional child birth with the strong design (follow up using Health belief model) will help policy maker to design appropriate interventions mechanism to increase institutional delivery.

Ethiopia’s Maternal Mortality Ratio (MMR), 676 per 100,000 live births, is among the highest in the world. Together with only five other countries, namely: India, Nigeria, Pakistan, Afghanistan, and the Democratic Republic of Congo, Ethiopia contributes about 50% to the total global burden.

Although there is significant progress in all developing regions, the average annual percentage decline of Maternal Mortality Ratio (MMR) in sub-Saharan Africa, of which Ethiopia contributes considerably, is only 1.7%. This is very much lower than the Millennium Development Goal (MDG) target of 5.5% [1,2].

Every pregnancy faces some risk which may not always be detected through the risk assessment approach during antenatal care. However, majority of deaths due to obstetric complications are preventable. So the best way to assure a safe and successful delivery outcome remains to be ensuring the presence of a skilled birth attendant at every child birth. The presence of a skilled birth attendant during delivery is also one of the indicators used in achieving MDG5. Surprisingly, in Ethiopia the proportion of births attended by skilled personnel is only 10% even in areas where women have access to the services [4]. However, it is not known why majority of pregnant women do not deliver their babies in a health institution.

## Methods

A community based follow up study was conducted in Debra Markos town from January17, 2012 to July 30, 2012. Debre Markos is the capital city of East Gojam zone located about 300 km from Addis Ababa, the capital city of Ethiopia. The town is divided into 7 kebeles (the smallest administrative unit) with a population estimated to be 62,469 (Census 2007). The town has one referral hospital, three health centers, three private clinics and one family guidance association clinic (FGA). All these institutions except the private clinics and FGA provide delivery service.

The study population was comprised of all pregnant women in Debre Markos, who were in their second and third trimester of pregnancy at the time of survey. Subjects were selected using simple random sampling technique from a list which included all second and third trimester pregnant women. The sample size was determined by using a single population proportion formula considering the following assumptions: magnitude of intention for institutional delivery 50%, (p = 0.5), 5% level of significance (α = 0.05). The final sample size was adjusted for none response rate of 10% and it was 422. All eligible pregnant women were initially asked about their preference regarding their place of delivery (home or health institution). These mothers were visited again after six months to check on their actual place of delivery.

Data was collected through face to face interview using a structured and pre-tested questionnaire while conducting house to house survey.

The data collection process had two phases. Phase I- Interview of pregnant women to assess their socio-demographic profile, preference about place of delivery and some factors associated with their choice of place for delivery. Phase II. Interview of mothers after they deliver: The pregnant women who had been interviewed in phase I were interviewed again to determine their actual place of delivery and associated factors for their choice.

Data was collected by 7 accelerated midwifery students from January 17, 2012 to July 30, 2012. Two midwives from Debra Markos Hospital supervised the data collection process.

Data was organized using EPI Info 2002 and exported to SPSS version 16.0 software package for analysis. Variables found significant (p–value ≤ 0.05) on bivariate analysis was included in multiple logistic regression analysis to determine the effect of various factors on the outcome variable and to control confounding effect. The results were presented in the form of tables, figures and text using frequency and summary statistics such as mean, standard deviation and percentage. These describes the study population in relation to the independent variables, like: age, education, income, age at first pregnancy, parity, any obstetric complication. Variables were categorized in the context of the constructs of the health belief model and other literature. The degree of association between the independent and dependent variables was analyzed using odds ratio with 95% confidence interval.

Ethical clearance was obtained from Institutional Review Board (IRB) of University of Gondar. A letter of cooperation was written for East Gojam Health Department and Debre Markos Wereda Health Office. Finally written consent was obtained from each study pregnant woman.

## Results

### Socio-demographic characteristics

Of the 422 pregnant women selected as sample, only 393 responded to the questionnaire, making the response rate of 93%. The mean age of the pregnant women was 28.26 years (28.26 ± 5.61 SD) years. Majority 319 (81.2%) of pregnant women were Amhara by ethnicity and 248 (63.1%) Orthodox Christian. Three hundred thirty six (85.5%) of the pregnant women reported to be married and 189 (48.1%) have attended secondary and high school education. With regard to their husband’s education, 311(79.1%) of them have at least secondary education (Table 1).

**Table 1:** **Socio-demographic Characteristics of mothers (N = 393) who are in their 2** ^**nd**^ **and 3** ^**rd**^ **trimesters of pregnancy in Debra markos town, East Gojam, July, 2012**

**Characteristics**	**Frequency(Percent)**
**Age of mothers during the interview (mean, SD = 28.26, ±5.610)**	
15-19	26(6.6)
20-24	72(18.3)
25-29	137(34.9)
30-34	84(21.4)
35-40	74(18.8)
**Marital status**	
Married	336(85.5)
Single	25(6.4)
Divorced/widowed	32(8.1)
**Religion**	
Orthodox	248(63.1)
Muslim	78(19.8)
Protestant	49(12.5)
Others*	18(4.6)
**Maternal educational status**	
No formal education	44(11.2)
Elementary	96(24.4)
Secondary &high school	189(48.1)
Collage and above	64(16.3)
**Ethnicity**	
Amhara	319(81.2)
Agaw	22(5.6)
Oromo	21(5.3)
Tigre	17(4.6)
Others**	14(3.3)
**Occupation**	
House wife	185(47.1)
Self-employee	78(19.8)
Gev-employee	74(18.8)
Students/daily workers	56(14.2)
**Husband educational status**	
No formal education	27(6.9)
Elementary	55(14.0)
Secondary & high school	109(27.7)
Collage and Above	202(51.4)

*Catholic, joba **Gurage, shinasha.

### Obstetrics characteristics

Majority 260 (66.2%) of the respondents become pregnant for the first time when they were 21–34 years old. For 153(38.9%) the current pregnancy is their first, sixty three (16%) of the pregnant women have faced at least one obstetric problem during the current pregnancy. 348(88.5%) have visited an antenatal clinic at least once. Out of 393 pregnant women who were interviewed after they delivered, 62.3% of them have delivered in health facilities. The rest of pregnant women delivered at home (Table 2).

**Table 2:** **Distribution of obstetrics characteristics of the pregnant women in D/M town, East Gojam zone, 2012**

**FACTORS**	**FREQUENCY (%)**
**Age at first pregnancy**	
≤20	130(33.1)
21-34	260(66.1)
≥35	3(0.8)
**Parity**	
0	153(38.9)
I-III	212(53.9)
≥IV	28(7.1)
**Child alive**	
0	167(42.5)
1-3	209(53.2)
≥4	17(4.3)
**Child died**	
0	312(79.4)
1	67(17.0)
2	14(3.6)
**Current ANC follow up**	
Yes	348(88.5)
No	45(11.5)
**Women’s actual place of delivery**	
Institution delivery(ID)	245(62.3)
Home delivery(HD)	148(37.7)
**Duration of labour for HD in hours (mean, SD = 8 hrs, 3.67)**	
≤3	20 (13.5)
3-24	122( 82.4)
>24	6 (4)
**Duration of labour for ID in hours (mean, SD = 16 hrs, 4.032)**	
≤3	6(2.4)
3-24	200(81.6)
>24	39(16)
**Mode of delivery in health institution(n = 245)**	
None instrumental vaginal delivery	147(60)
Instrumental vaginal delivery	66(26.9)
Cesarean section	32(13.1)
**Attendants for home delivery(n = 148)**	
TBA	81(54.7)
HEW	37(25)
Family members	24(16.2)
No attendant/Self-attendance	6(4.1)

**Figure 1:**
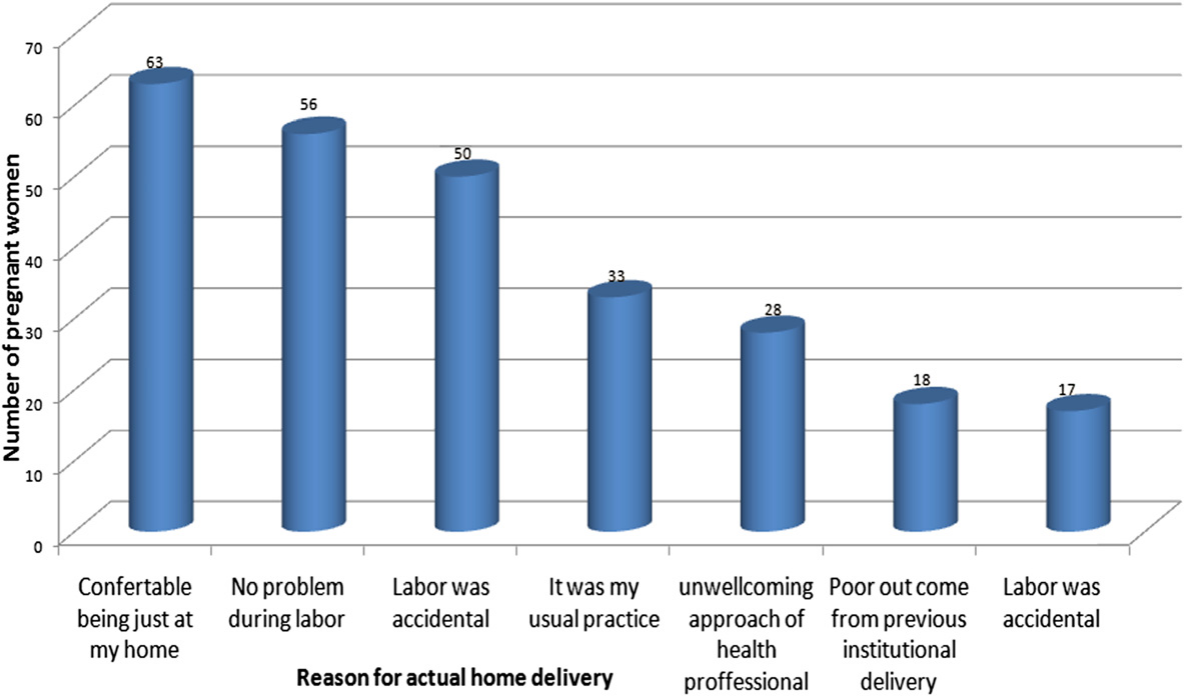
**Reasons for Home delivery among mothers who were in their second and third trimesters in Debra-Markos town, east Gojam, northern Ethiopia, July, 2012.**

Different reasons were given by the mothers for home delivery. The most frequently reported reason was being comfortable at home which was 63 (16%) mothers, followed by delivery at home is my usual practice 56(14.2%) and others (Figure 1).

### Intention (preference) about place of delivery

**Figure 2:**
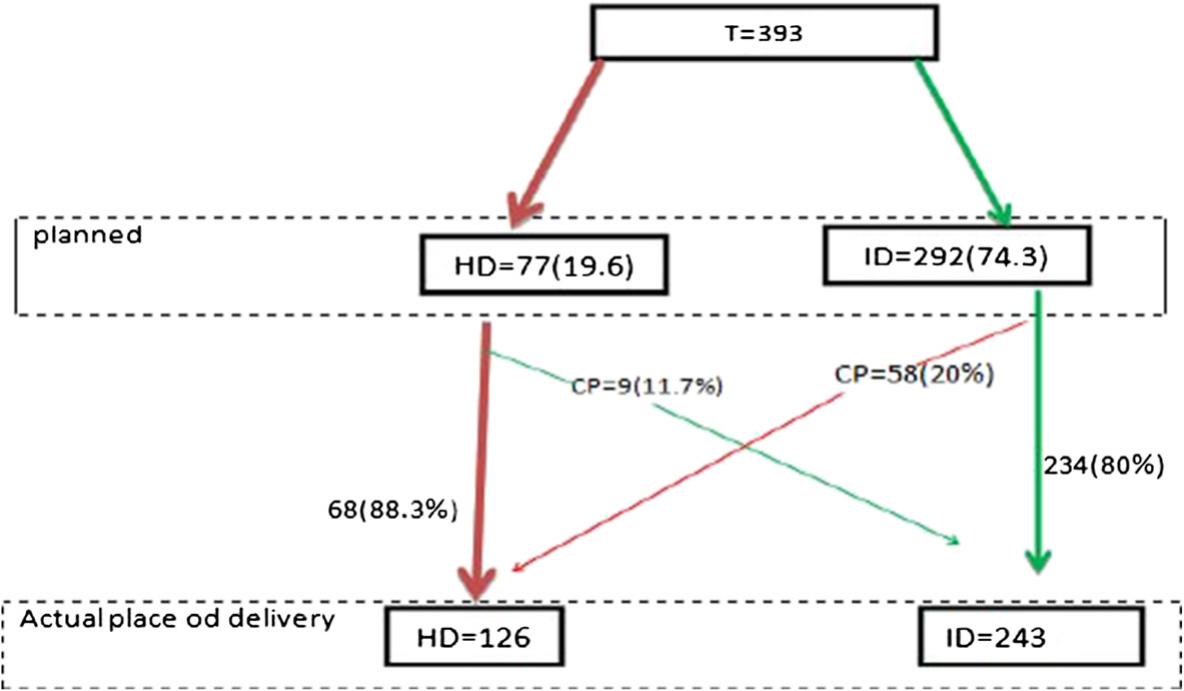
**Planned and actual place of delivery among mothers who were in their second and third trimesters in Debre-markos town, east Gojam, northern Ethiopia, July, 2012.**

The study results revealed that among the 393 pregnant women 292 (74.3%) preferred to deliver their current pregnancy in a health facility, 77 (19.6%) preferred to deliver in their home, while the rest 24(6.1%) have not yet decided where to deliver. These women were revisited after six month (after they all delivered). Two hundred thirty four 234(80%) of those who previously planned for institutional delivery, actually delivered in health institution while 58 (20%) of them ended up with home delivery. On the other hand, of the 77(19.6%) of the pregnant women who planned to have a home delivery 9 (11.7%) of them ended up with institutional delivery (Figure 2).

Fifty eight (20%) women were asked the reason why they changed their planned place of delivery from health institution to their home and the reported reasons include: labor is accidental and no problem has occurred during labor at home.

### Distribution of predisposing, enabling, reinforcing factors, women’s intention and decision making power

Three hundred (93.2%) of the respondents knew at least one advantage of institutional delivery. But only 198 (50.4%) have good attitudes towards institutional delivery.

Regarding their perceptions about pregnancy and child birth complications as well as importance of getting skilled help at child birth, only 279 (71.75%) of the women feel that they may be susceptible to develop delivery complications which can be hazardous to their health, but 204(73.1%) of them agreed that if they get a skilled attendant during delivery, it will be beneficial to their health and the health of their newborns.

According to the beliefs of the study participants, majority 288 (73.3%) of their husbands prefer institutional delivery. Moreover, 292 (74.3%) of the pregnant women intended to deliver in institutions. Regarding women’s decision making power in relation to getting institutional delivery services, 291(74.0%) can make this decision by themselves, while the rest need to get the decision either from their husbands or their relatives (Table 3).

**Table 3:** **Distribution of predisposing, enabling, reinforcing factors, women’s intention and decision making power in Debra-Markos town, East Gojam zone, 2012**

**FACTORS**	**FREQUENCY (%)**
**Knowledge about ID service**	
Knowledgeable	366(93.2)
Not knowledgeable	27(6.8)
**Attitudes towards ID**	
Favorable	198(50.4)
Unfavorable	91(23.2)
Indifferent	104(26.5)
**Perceptions about threat of delivery complication**	
Agree	279 (71.75)
Disagree	56 (13.5)
Indifferent	58(14.75)
**Perception about benefits of getting SDA**	
Agree	204 (73.1)
Disagree	41 (14.69)
Indifferent	34 (12.18)
**Perception about barrier to get SDA**	
Yes	32 (11)
No	247 (89)
**Husbands preference to place of delivery**	
ID	288 (73.3)
HD	70 (17.8)
Do not know	35 (8.9)
**Women’s intentions about place of delivery**	
ID	292 (74.3)
HD	77 (19.6)
Not yet	24(6.1%)
**Final Decision maker**	
The woman herself	291 (74.0)
Husband/partner	47 (12.0)
Relatives	43 (10.9)

### Factors associated with actual delivery place of the women

Maternal age, the pregnant women’s and their husbands educational status, women’s perceptions about delivery complications and benefits of institutional delivery, obstetric problems during current pregnancy or child birth, antenatal care follow up and their intention for place of delivery were significantly associated with actual place of delivery (P-values < 0.05).

Pregnant women in the age range of 15–19 years are about 5 times more likely to deliver in Health facilities when compared to those between 35–39 years (AOR = 4.84, 95% CI = 1.56-12.64) and pregnant women who have at least attended college are 12 times more likely to deliver in a health facility compared to those who have no formal education (AOR = 12.51, 95% CI = 1.08 -14.56), On the other hand pregnant women whose husbands attended college education or higher are 3 times more likely to deliver in health institutions compared to those whose husbands had no formal education(AOR = 3.01, 95% CI = 1.09-8.02).

Pregnant women who have receptive perception about pregnancy and delivery complication are 3 times more likely to deliver in a health institution compared to those who have non-receptive perception (adjusted AOR = 3.21, 95% CI = 1.26-8.16) and women who have receptive perception about the benefits of institutional delivery are about 2 times more likely to deliver in health institution than those who have non-receptive perception (AOR = 1.95, 95% CI = 1.52-3.04).

Pregnant women who attended ANC follow up during the current pregnancy, are about 2 times more likely to deliver in health facility compared to pregnant women who were not attending ANC (AOR = 1.98, 95% CI = 1.02-3.39).

Pregnant women who faced at least one obstetric problem were about 4 times more likely to give institutional delivery compared to those who have not faced any problem (AOR = 4.33, 95% CI = 1.26-14.83). Current intention of pregnant women about place of delivery is another strong predictor of the place of delivery. Women who intended to give institutional birth were 7 times more likely to deliver in health institution compared to those who intended to deliver at their home (AOR = 7.03, 95%CI = 3.045-10.23) (Table 4).

**Table 4:** **Bivariate and multiple logistic regression analysis of factors associated with institutional delivery service utilization among pregnant mothers in Debre-markos town, July- 2012**

**Factors**	**HM**	**ID**	**Crude ORs(95% CI)**	**Adjusted ORs(95% CI)**
Age of pregnant women				
15- 19	10	16	4.038 (1.581-10.316)	4.836(1.562-12.641)
20-24	20	52	6.562 (3.187-13.509)	7.026(0.0541-18.033)
25-29	28	109	9.825 (5.108-18.897)	2.501(0.831-7.525)
30-34	37	47	3.206 (1.650-6.228)	1.626(0.524-5.047)
35-39	53	21	1	1
Maternal Educational status				
No formal education	25	19	1	1
Primary education	51	45	1.161(0.566-2.382)	3.038(0557–16.561)
Secondary and high school	48	141	3.865(1.957-7.633)	4.685(.558-39.3711)
Collage and above	24	40	2.193 (1.003-4.795)	12.508(1.082-14.557)
Women’s occupation				*
House wife	92	93	1	
Self-employee	10	68	6.727 (3.262-13.871)	
Gev-employee	16	58	3.586 (1.921-6.693)	
Husband education				
No formal Education	15	12	1	
Primary education	31	24	0.968(.383-2.447)	
Secondary and high school education	47	62	1.649(.706-3.852)	
Collage and above	85	147	3.341(1.472-7.585)	3.013(1.085-8.021)
Age at first pregnancy				*
<20	62	68	1	
21-34	85	175	1.877(1.220-2.888)	
≥35	1	2	1.824 (0.161-2.610)	
Parity				*
0	45 + 3	105	5.930(2.307-15.245)	
1-3	81	131	8.800(3.345-23.153)	
≥4	22	6	1	
Knowledge about ID service				*
Poor	56	92	1	
Good	92	232	4.863 (5.671-20.807)	
Perceptions about threat of obstetric complication				
None receptive	93	45	1	1
Receptive	55	200	7.515(4.723-11.958)	3.208(1.262-8.155)
Perceptions about benefits to have ID				
Non receptive	110	91	1	1
Receptive	38	154	4.899 (3.121-7.689)	1.952(1.515-3.042)
Women’s Decision making				*
No	50	43	1	
Yes	96	202	2.447 (1.522-3.933)	
ANC follow up				
No	31	13	1	1
Yes	116	232	4.769 (2.404-9.460)	1.975(1.021-3.392)
Obstetric problems				
No	135	195	1	1
Yes	13	50	2.663 (1.392-5.093)	3.21 (1.261-8.163)
Intentions about place of delivery				
HM	68	9	1	1
ID	60	232	11.320(3.426-37.408)	7.032(3.045-10.234)

*= Not significantly associated on backward logistic regression.

## Discussion

The study revealed that among 393 pregnant women 292(74.3%) preferred to deliver in a health facility while 77(19.6%) preferred to deliver at home.

The current intention for institutional delivery (74.3%) is less than expected value when compared to the findings from same study done in Jima town in 2005 [4]. This may be due to difference in the subjects of the study. The former study included only third trimester pregnant women, while the current study included second trimester pregnant women. Pregnant women who are in their second trimester have yet to determine their place of delivery. This could have reduced the proportion of pregnant women who intended to have institutional delivery.

The finding of this study shows a higher percentage of pregnant women who preferred institutional delivery when compared to a study done in Switzerland. The difference could be mainly due to the difference in a model of delivery service. In the Ethiopian context home delivery is Model 1, where lay providers recognize complications occurring during delivery and the family organizes access to a facility with essential obstetrics care (EOC). In some developed countries like Switzerland, home delivery is Model 2, where labor is attended at home by professionals who recognize complications and family or provider organizes, referral to EOC [5,6].

From this prospective follow up study it was found that maternal age is another important predictor of institutional delivery service utilization. Pregnant women who are in the age range of 15–19 years are about 5 times more likely to give birth in health institutions compared to pregnant women with age of 35 and above (AOR = 4.84, 95% CI = 1.56-12.64). This finding is consistent with studies done in other parts of Ethiopia (Sekela district, Munesa wereda, North Gondar and Metekel zone) which shows younger women are more likely to deliver in a health facility as compared to older ones [7-11]. The explanation for this could be that younger women are more likely to utilize modern health care facilities than older women as they have greater exposure and knowledge modern health care, because they have more access to modern education.

The present study shows that amongst the maternal characteristics, educational status has the strongest association with the use of institutional delivery service. Pregnant women with higher level of education (college and above) were 12 times more likely to deliver in a health facility compared to those who have no formal education (AOR = 12.51, 95% CI = 1.08-14.56). This finding is in agreement with studies conducted in different parts of Ethiopia [7-11]. Similar finding reported from studies done in Nigeria, Pakistan and Kenya showed significant association of maternal education with the use of institutional delivery services [12-15].

Another finding of the present study is that the ANC visit is also an important predictor of institutional delivery service utilization. Pregnant women who have ANC visits during the current pregnancy are 2 times more likely to deliver in a health facility compared to those who do not have ANC visits (AOR = 1.98, 95% CI = 1.02-3.39). This result is consistent with the findings of a study done in Sekela district where those who had ANC attendance were 4 times more likely to deliver in health facilities compared to those who did not have ANC visits [7]. This may be because of the information as well as the experiences they have gathered during the follow up visits which could have influenced their decision to deliver in health facilities. Another possible explanation may be that ANC follow up by itself is one of maternal service utilization, so that factors which influence utilization of ANC might have influenced institutional delivery service utilization in a similar manner.

Pregnant women’s perception about susceptibility to delivery complications, severity of delivery complications and benefit of institutional delivery is another critical factor in predicting probability of institutional delivery. Pregnant women who have receptive perception about threat of delivery complication are about 3 times more likely to deliver in health institutions compared to those who have none receptive perception (AOR = 3.21, 95%CI = 1.26-8.16). This finding is similar with the findings of a study done in Bangladesh where the perceived complication of child birth was the most common reason for institutional delivery [16]. Pregnant women in Munesa wereda who were knowledgeable about child birth complication and their susceptibility to these complications were 2 times more likely to deliver in health institution than those who were not knowledgeable [8].

In the present study, it was found that pregnant women who have receptive perception about the benefits of institutional delivery are 2 times more likely to deliver in a health facility compared to those who have non-receptive perception. This result is in conformity with a study conducted in Tanzania where perceived benefits of the importance of institutional delivery increased the odds of delivering in a facility by 2 folds among multiparous women [17]. This may be due to the fact that, perceived susceptibility and severity of specific health problem are more powerful in promoting preventive health care-seeking behavior. The greater the perceived susceptibility and severity of health problems, the greater the care-seeking preventive behaviors [18]. Therefore awareness about the risks and severity of specific childbirth complications, and the benefits of institutional delivery service utilization which are acquired from ANC follow up tend to increase preventive care-seeking.

The intention of pregnant women about place of delivery, which has the highest odds as a predictor of institutional delivery in the present study has strong association with their actual place of delivery. Pregnant women who intended to give birth in a health institution are 7 times more likely to have institutional delivery than those who intended to have home delivery (AOR = 7.03, 95% CI = 3.05-10.23). This result is consistent with the studies done in Switzerland, Syria, Kenya and different parts of Ethiopia which revealed that pregnant women who preferred to deliver at home were more likely to use unsafe delivery practice compared to those who intended to deliver at a health facility [6,8,19,20]. This is because intention has the most immediate influence on behavior. If a person intends to perform a behavior, then it is more likely he or she will do so and if the person doesn’t intend to perform it, then the behavior is unlikely to be performed [18].

Finally this follow up study tried to correct the limitations of cross sectional researches by reducing a recall period only to a maximum of 6 months and measuring the underlying factors while the women were still pregnant and by following them until they deliver to assess their actual delivery practices. On the other hand the limitation of the current study is that it involved only urban women while most of the reported maternal deaths is found in rural areas of the country.

## Conclusions

In conclusion, this study revealed that the proportion of pregnant women, who intended to give birth in health facility, is low.

Common reasons given by mothers for giving birth in their home were just comfortable being at home, no problem has occurred during labour, delivering at home is their usual practice and labour was accidental.

Maternal age, the educational status of both the women and their husbands, status of ANC follow up, intention about place of delivery, perception about susceptibility, severity of delivery complication and perceived benefits of institutional delivery are important predictors of the actual place of delivery. Increasing awareness of mothers about their susceptibility to unexpected severe obstetric complication and benefits of institutional delivery services are recommended.

## References

[1] WHO (2010). Number of Maternal Deaths Declines Worldwide; Regional, Country Challenges Remain.

[2] Hogan M, Foreman K, Naghavi M, Ahn S, Wang M, Makela S (2010). Maternal mortality for 181 countries, 1980–2008: systematic analysis of progress towards MDG5. Lancet.

[3] Central Statistical Agency [Ethiopia] and ORC Macro (2011). Ethiopia Demographic and Health Survey.

[4] Ayalew B. Factors That Influence Intention of Pregnant Women for Their Delivery and the Factors That Determine Actual Delivery Service Utilizations in Jimma Town [un Published Mastern Thesis, Addis Abeba University]

[5] Koblinsky A, Campbell O, Heichelheim J (1999). Organizing delivery care: what works for safe motherhood?. Bull World Health Organ.

[6] Liebrich A, Voegeli T, Giunter K, Kunz I, Ziillig M, Schindler C (1996). Home versus hospital deliveries: follow up study of matched pair for procedures and outcome. BMJ.

[7] Teferra AS, Alemu FM, Woldeyohannes SM. Institutional delivery service utilization and associated factors among mothers who gave birth in the last 12 months in Sekela District, North West of Ethiopia: A community-based cross sectional study. BMC pregnancy and childbirth 2012;1(12):74-85. 10.1186/1471-2393-12-74 PMC3449175 22849421

[8] Amano A, Gebeyew A, Berhanu A. Institutional delivery service utilization and associated factors among mothers who gave birth in the last 12 months prior to the study in munesa wereda, Arsi zone 2011. BMC Pregnancy and child birtt. 2012(12):105-116.

[9] Nigussie M, Haile Mariam D, Mitike G (2004). Assessment of safe delivery service utilization among women of childbearing age in North Gondar Zone, North West Ethiopia. Ethiop J Health Dev.

[10] Tura G, G/Mariam A (2008). Safe delivery service utilization in Metekel Zone, North West Ethiopia. Ethiop J Health Sci.

[11] Malkamu A. Assessment of factors affecting utilization of maternal health care services in Assaita and Dubti towns, afar regional state [masters thesis]. In press 2010.

[12] Dhakal S, Teijlingen E, Raja E, Dhakal K (2011). Skilled care at birth among rural women in Nepal: practice and challenges. J Health Popul Nutr.

[13] Ikeako L, Onah H, Iloabachei G (2006). Influence of formal maternal education on the use of maternity services in Enugu, Nigeria. J Obstet Gynecol.

[14] Agha S, Carton TW (2011). “Determinants of institutional delivery in rural Jhang, Pakistan.”. Int J Equity Health.

[15] Ochako R, Fotso J, Ikamari L, Khasakhala A (2011). Utilization of maternal health services among young women in Kenya: insights from the Kenya Demographic and Health Survey, 2003. BMC Pregnancy Childbirth.

[16] Choudhury N, Moran A, Alam M, Zunaid K, Sabina A, Rashid F (2012). Beliefs and practices during pregnancy and childbirth in urban slums of Dhaka, Bangladesh. BMC Pregnancy Childbirth.

[17] Ndao S, Mbaruku G, EKruk M (2012). Parity and institutional delivery in rural Tanzania: a multilevel analysis and policy implications. Health Policy Plan.

[18] Daniel E, Danulta K. Theory of Reasoned Action, Theory of Planned Behavior, and the Integrated Behavioral Model. in: Foreword by C, Tracy O. Health Behavior and Health Education. San Francisco: .Jossey-Bass A Wiley Imprint, 2008: 67-96.

[19] Bashour H, Abdul Salam A (2005). Syrian women’s preferences for birth attendant and birth place. PMC.

[20] Ableru J (2003). Care-Seeking During Pregnancy, Delivery, and the Postpartum Period: A Study in Homabay and Migori Districts.

